# The Prognostic Value of CD206 in Solid Malignancies: A Systematic Review and Meta-Analysis

**DOI:** 10.3390/cancers13143422

**Published:** 2021-07-08

**Authors:** Jens M. Debacker, Odrade Gondry, Tony Lahoutte, Marleen Keyaerts, Wouter Huvenne

**Affiliations:** 1 Department of Head and Skin, Ghent University, 9000 Ghent, Belgium; Wouter.Huvenne@UZGent.be; 2 Department of Head and Neck Surgery, Ghent University Hospital, 9000 Ghent, Belgium; 3 Department of Nuclear Medicine, University Hospital Brussels, 1090 Brussels, Belgium; Odrade.Gondry@vub.be (O.G.); Tony.Lahoutte@vub.be (T.L.); Marleen.Keyaerts@vub.be (M.K.); 4 In Vivo Cellular and Molecular Imaging, Vrije Universiteit Brussel, 1090 Brussels, Belgium

**Keywords:** tumor associated macrophages, CD206, macrophage polarization

## Abstract

**Simple Summary:**

The role of innate immune cells in the tumor microenvironment (TME), more specifically the presence of the tumor associated macrophages (TAMs), is becoming more important in the prognosis and treatment of patients diagnosed with malignancies. The aim of this systematic review and meta-analysis was to assess the potential prognostic value of CD206-expressing TAMs, a subclass of macrophages, which were previously proposed to negatively impact the patient’s prognosis. We identified 27 manuscripts describing the role of CD206 in patient prognosis for 14 different tumor types. Despite a large heterogeneity in the results, we identified a significantly worse overall and disease-free survival for patients with increased CD206-expressing TAMs in the TME. The use of CD206-expressing TAMs could therefore be used as a prognostic marker in patients diagnosed with solid malignancies.

**Abstract:**

An increased presence of CD206-expressing tumor associated macrophages in solid cancers was proposed to be associated with worse outcomes in multiple types of malignancies, but contradictory results are published. We performed a reproducible systematic review and meta-analysis to provide increased evidence to confirm or reject this hypothesis following the Preferred Reporting Items for Systematic Reviews and Meta-analyses statement. The Embase, Web of Science, and MEDLINE-databases were systematically searched for eligible manuscripts. A total of 27 papers studying the prognostic impact of CD206 in 14 different tumor types were identified. Meta-analyses showed a significant impact on the overall survival (OS) and disease-free survival (DFS). While no significant differences were revealed in progression-free survival (PFS) and disease-specific survival (DSS), a shift towards negative survival was correlated with increased CD206-expresion. As a result of the different tumor types, large heterogeneity was present between the different tumor types. Subgroup analysis of hepatocellular carcinoma and gastric cancers revealed no heterogeneity, associated with a significant negative impact on OS in both groups. The current systematic review displays the increased presence CD206-expressing macrophages as a significant negative prognostic biomarker for both OS and DFS in patients diagnosed with solid cancers. Because a heterogenous group of tumor types was included in the meta-analysis, the results cannot be generalized. These results can, however, be used to further lead follow-up research to validate the specific prognostic value of CD206 in individual tumor types and therapeutic approaches.

## 1. Introduction

The role of the tumor microenvironment and the presence of immune cells is becoming increasingly important in the diagnosis and treatment of patients with solid cancers [1,2]. The presence of tumor infiltrated lymphocytes (TIL) shows an important favorable prognostic factor in several solid tumors, while the presence of tumor associated macrophages (TAM) was associated with both positive and negative prognosis, depending on the predominant subtype of represented macrophages [3,4]. 

TAMs form a heterogeneous group of cells, explaining the differences in the prognostic value when only looking at the amount of macrophages in the tumor [3]. Historically, a division was made between M1-macrophages and M2-macrophages where the M1-type macrophages were characterized by their proinflammatory role, inducing tissue damage and inhibiting tumor cell proliferation [5]. Consequently, M1-macrophages are thought to have an antitumorigenic effect and, similar to TIL-density, can be a general positive prognostic factor. In contrast, the M2-macrophages are typically identified in wound healing sites, providing growth factors to increase tissue healing. Moreover, in oncological settings, M2-macrophages decrease the presence of other immune cells by producing protumoral growth factors associated with worse patient survival [6]. The balance between the anti- and protumorigenic macrophages can therefore have an important impact on the prognosis of patients diagnosed with malignancies.

However, novel insights showed that this M1/M2 division is outdated, and macrophages are rather characterized by a spectrum of subtypes [7]. Within these TAMs, the CD206-expressing macrophages were shown to be increased in murine hypoxic tumors and appear to be an independent risk factor for response to systemic therapies in patients diagnosed with solid cancers [8,9]. The presence of CD206-expressing macrophages could hence be used as a tool to tailor future oncological therapies. However, the specific prognostic value of CD206 expression in solid tumors remains unapprehended. 

The purpose of the current systematic review and meta-analysis was to identify all manuscripts describing the prognostic value of CD206-expression in human solid tumors to gather all current knowledge on CD206 as a prognosticator on the patient’s survival.

## 2. Materials and Methods

The current systematic review was conducted according to the Preferred Reporting Items for Systematic Reviews (PRISMA) guidelines and the Cochrane guidelines for prognostic reviews [10,11]. The review protocol was registered before review initiation on the International prospective register of systematic reviews (PROSPERO) with registration number CRD42020192849.

### 2.1. Development of a Search String

The PICOTS (patient-population, intervention, comparator, outcoming, timing & setting) framework was used to develop a study-specific search string, which was based on the research question: ‘What is the prognostic value of CD206-expression in the tumor microenvironment of solid malignancies?’ This resulted in the development of a search string which used synonyms of the following sentence: “CD206 AND prognostic factors AND cancer”. However, an important synonym of CD206 is ‘macrophage mannose receptor’ or abbreviated into ‘MMR’. Because the abbreviation ‘MMR’ is an acronym for a lot of other important medical terms, such as ‘mismatch repair’ and the ‘measles, mumps and rubella’-vaccine, we added a filter to remove the most important acronyms of MMR. This resulted in the sentence ‘(CD206 OR (MMR NOT (other acronyms of MMR)) AND prognostic factor AND cancer”. The sentence was hence translated into a search string for NLM PubMed, Ovid Embase, and Clarivate Analytics Web of Science. The used search strings and the individual hits on the date of search are given in Table A1, Table A2 and Table A3.

### 2.2. In-and Exclusion Criteria

We included both prospective and retrospective longitudinal studies investigating the impact of CD206-expression on the oncological outcome of patients diagnosed with solid cancers, specifically excluding hematological malignancies. The expression of CD206 on the tumor or in the microenvironment should be provided using a quantitative or semiquantitative methodology. The amount of CD206-expression should be associated directly with the patient’s survival data. No exclusions were performed based on the treatment prior to or following the tissue extraction. Reviews and meta-analysis were excluded. No manuscripts were excluded based on the follow-up period.

### 2.3. Data Collection 

After initial search, all hits were imported into EndNote (Clarivate Analytics, Philadelphia, PA, USA). Duplicates were removed using the built-in duplicate tool, and manually screened. Two independent reviewers (J.D.&O.G.) assessed both titles and abstracts of the retrieved records from the original data search using the Rayyan tool for systematic reviews [12]. All potentially relevant studies were assessed independently for eligibility criteria. When any doubt remained after title and abstract-assessment, the record was included for full-text assessment. After assessing all titles and abstracts, full texts were assessed for eligibility criteria. When final inclusions were agreed on, all data was manually extracted. The extracted data included the first author, the geographical location of the study, the sample size, the tumor characteristics, the used treatment modalities, the used IHC antibody, and the prognostic outcomes. Prognostic outcomes were defined as overall survival (OS), disease-free survival (DFS), disease-specific survival (DSS), progression-free survival (PFS), metastasis-free survival, and/or locoregional control.

### 2.4. Data Analysis

The hazard ratios (HR) and their corresponding 95% confidence intervals (CI) of the individual oncological outcomes were used to compare the impact of CD206-expression on the patient’s outcome. Hazard ratios had to compare high CD206-expression over low CD206-expression; hence, HR > 1 indicated worse survival associated with increased CD206-expressing tumors. When the HR and its corresponding 95% CI were not reported, we used the method as described by Tierney et al. to estimate the HR based on the described data [13]. When both uni- and multivariable analysis were performed on the same prognostic factor, the multivariable HR as reported by the author was used for meta-analysis. When insufficient data in terms of prognostic outcome were described in the manuscript, the original authors were contacted. If no response was received after 2 reminders within one month, the article was excluded. Meta-analysis was performed in Review Manager 5.4 (The Cochrane Collaboration), comparing the different HRs per oncological outcome.

## 3. Results

### 3.1. Search Results and Study Characteristics

**Figure 1:**
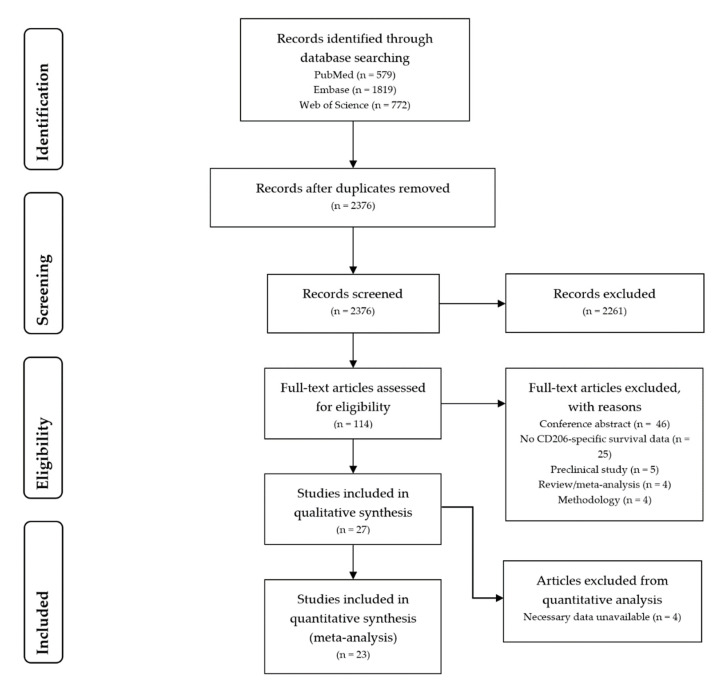
Flow diagram of literature search and selection.

Searches on the PubMed, Embase, and Web of Science databases on the 15 July 2020 led to the identification of 3170 hits. A total of 795 duplicates were identified and removed, resulting in 2375 unique hits. After evaluating titles and abstracts 114 records were included for full-text assessment. Eventual screening of the full texts led to the inclusion of 27 manuscripts for data-extraction. Figure 1 displays the PRISMA-flow chart detailing the process of manuscript identification and selection [10].

All manuscripts were published between 2012 and 2020. Appendix A Table A4 summarizes the study characteristics of the included studies and their associated hazard ratios. As all types of solid tumors were included, a total of 14 different cancer types were identified describing the impact of CD206-expression on survival: breast cancer (*n* = 1), colo(rectal) cancer (*n* = 3), ovarian cancer (*n* = 2), esophageal cancer (*n* = 1), gastric cancer (*n* = 3), glioma (*n* = 1), hepatocellular carcinomas (*n* = 5), cholangiocarcinoma (*n* = 1), head and neck malignancies (*n* = 3), pancreatic cancers (*n* = 3), penile cancer (*n* = 1), prostate cancer (*n* = 1), kidney cancer (*n* = 1), and melanoma (*n* = 1). Immunohistochemistry or immunofluorescence were used for the analysis of all studies. Various CD206-targeting antibodies were reported in the manuscripts, and if available, the clone is given in Appendix A Table A4. No coherent methodology for CD206-quantification was described. Two main methodologies were described for (semi-)quantification: (1) quantification of the CD206-expressing cells and splitting in high and low expressing cells based on the median, and (2) semiquantification on a scale from 0–3.

Three of the included manuscripts did not have sufficient data in the manuscript for quantitative data extraction [14,15,16]. Because no response was obtained from the corresponding authors one month after requesting extra information, these manuscripts were excluded from final meta-analysis. 

### 3.2. Meta-Analysis

**Figure A1:**
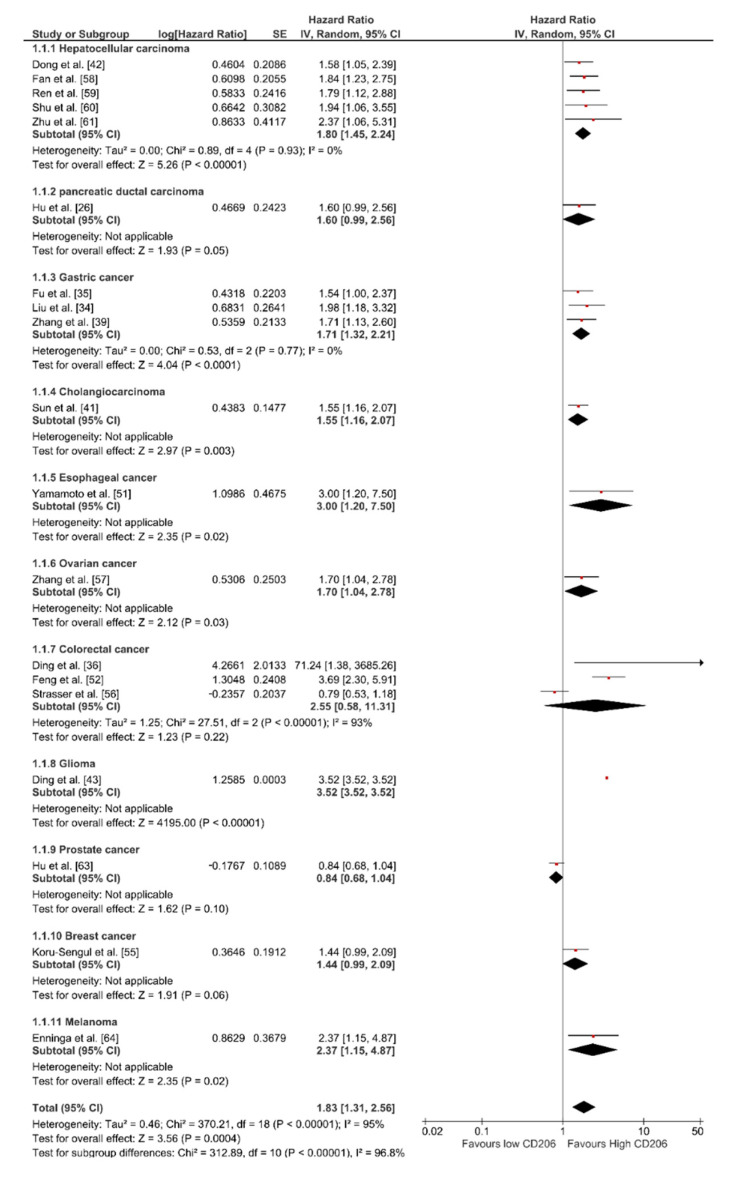
Forrest plot of impact of CD206-expression on overall survival of patients diagnosed with solid cancers. SE: standard error; IV: Interval variable; CI: confidence interval; df: degrees of freedom.

**Figure A2:**
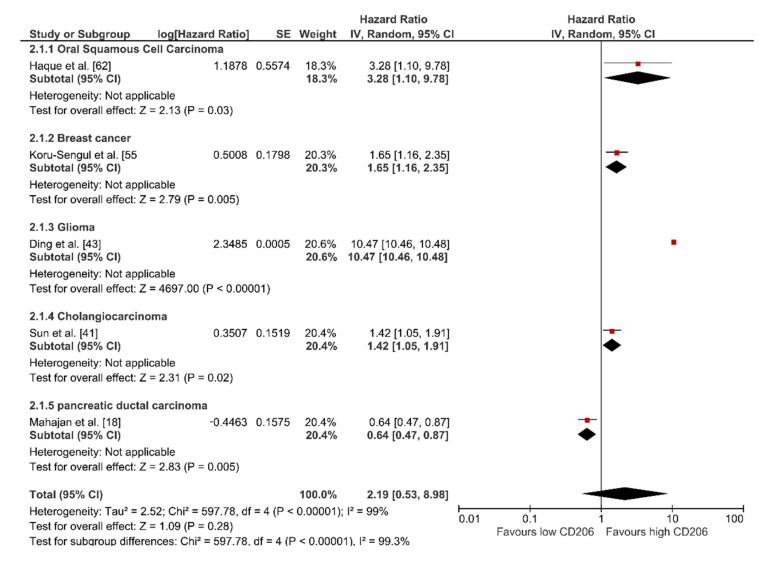
Forrest plot of impact of CD206-expression on the progression-free survival of patients diagnosed with solid cancers. SE: standard error; IV: Interval variable; CI: confidence interval; df: degrees of freedom.

**Figure A3:**
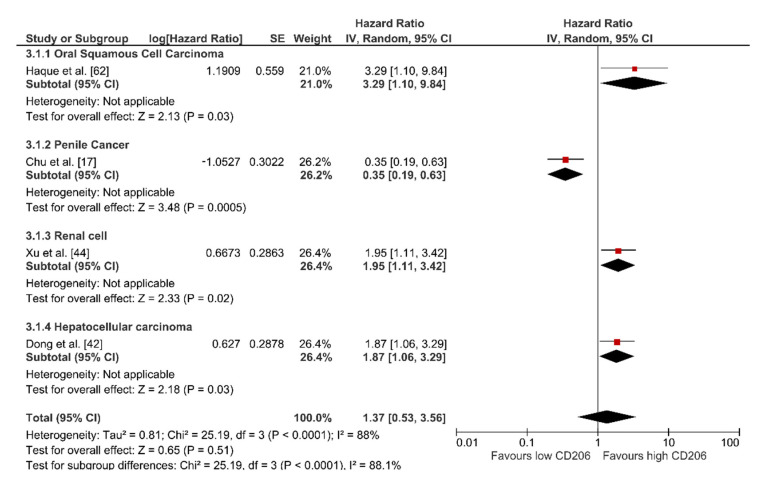
Forrest plot of impact of CD206-expression on disease specific survival of patients diagnosed with solid cancers. SE: standard error; IV: Interval variable; CI: confidence interval; df: degrees of freedom.

**Figure A4:**
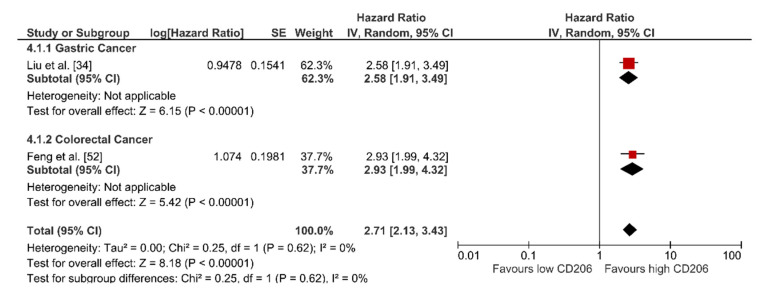
Forrest plot of the impact of CD206-expression on the disease-free survival of patients diagnosed with solid cancers. SE: standard error; IV: Interval variable; CI: confidence interval; df: degrees of freedom.

Meta-analysis was performed on the overall, progression-free, disease-specific, and disease-free survival of the included manuscripts (as illustrated in Appendix B Figure A1, Figure A2, Figure A3 and Figure A4). 

#### 3.2.1. Overall Survival

For OS, a total HR of 1.83 [1.31–2.56] was found, showing a significant impact on survival as a result of CD206-expression. However, as might be expected from the wide inclusion criteria, a considerable heterogeneity (I^2^ = 95%) was found between the results of the different studies. This heterogeneity disappeared in two out of three cancer types where multiple studies discussed OS for the same tumor type. In hepatocellular carcinomas, a HR of 1.80 [1.45–2.24] and in gastric cancer a HR of 1.78 [1.37–2.3] were identified with no associated heterogeneity (I^2^ = 0%). In contrast, colorectal cancers showed a nonsignificant result of 2.55 [0.58–11.31], with considerable heterogeneity (I^2^ = 93%).

#### 3.2.2. Progression-Free, Disease-Specific, and Disease-Free Survival

For PFS (*n =* 5), DSS (*n* = 4), and DFS (*n* = 2), no subgroup analysis was possible, as per subgroup only one study was included. This resulted in a respective HR [95%CI] of 2.19 [0.53–8.98], 1.37 [0.53–3.56], and 2.71 [2.13–3.43]. These results indicate a nonsignificant shift towards a worse prognosis on the PFS and DSS as a result of CD206-expression. However, I^2^ was 95% and 99% for PFS and DSS, respectively. Interestingly, the study by Chu et al. (2020) and Mahajan et al. (2018) were the only studies to describe a significant positive impact of CD206-expression on DSS and PFS, respectively [17,18]. In contrast, the two studies describing a HR for DFS do show a significant impact of CD206-expression with a I^2^ of 0%.

## 4. Discussion

In the current review, we systematically collected all studies that correlated CD206-expression in solid cancers with oncological outcomes. We identified a total of 27 studies in 14 different tumor types of which 23 performed statistical analysis to identify the prognostic impact between high and low-expression of CD206 in these tumors. To the best of our knowledge, we here describe the first meta-analysis showing CD206-expression as an independent prognosticator for OS, and with less certainty DFS, in multiple types of solid cancer. While no significant impact was found, our results also indicated a shift towards worse DSS and PFS in patients with increased CD206-expression.

**Figure 2:**
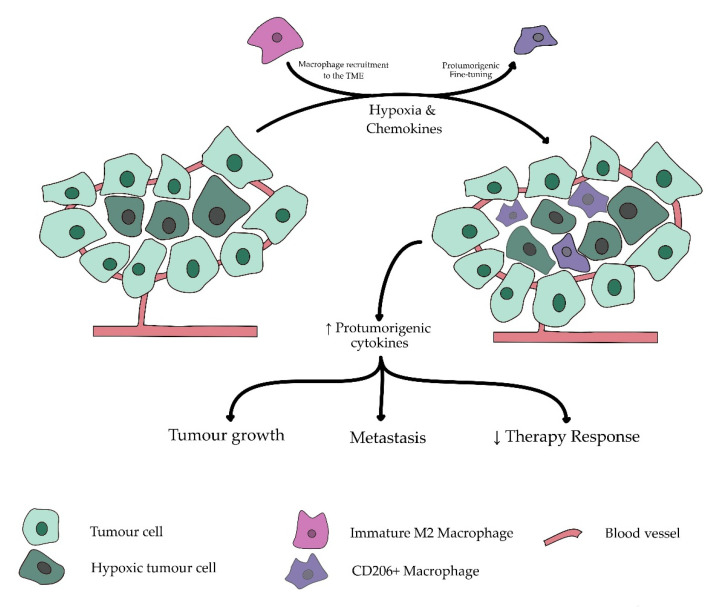
Simplified overview of the role of CD206+ macrophages in hypoxia-induced tumor progression. Tumor growth and insufficient coexpanding blood vessels often result in hypoxic regions in solid malignancies. In these hypoxic regions, a switch is made to anaerobic glycolysis, inducing production of inflammatory metabolic lactates. Consequential increased inflammation attracts macrophages to tumor microenvironment by differentiating circulating monocytes to macrophages and attracting tissue resident macrophages. Under influence of hypoxic environment, these macrophages are further fine-tuned by multiple chemokines developed by tumor cells and its microenvironment to suppress local inflammation and decrease attraction of antitumorigenic immune cells while developing additional protumorigenic cytokines. These protumorigenic effects result in an increased risk for metastasis, decreased therapy response, and further enhance tumor growth.

An upregulation of CD206 is typically described on the so-called ‘M2′-or ‘alternatively activated’-macrophages which are more commonly found in the hypoxic regions of solid cancers [19,20,21]. The presence of hypoxia is a known negative prognosticator in multiple types of solid cancer. As illustrated in Figure 2, its association with CD206-expressing macrophages could thus contribute to its underlying prognostic process [22,23,24]. While not limited to hypoxic regions, the presence of hypoxia in the tumor microenvironment was able to attract and polarize macrophages into the protumorigenic M2 subtype [25]. This process is based on the increase of the anaerobic metabolite lactate in the hypoxic regions of the tumor, causing an inflammatory response, hence attracting the anti-inflammatory macrophages [26,27]. As a result of the hypoxia, these macrophages are then fine-tuned to express multiple protumorigenic cytokines and growth factors such as EGF and VEGF, promoting tumor vascularization and growth, resulting in increased metastases and worse survival [28,29].

However, CD206 is not the only marker associated with protumorigenic TAMs. Other markers are more commonly used to differentiate these protumorogenic M2-macrophages, of which CD163 is the most common. Parallel with our results, an increased expression of CD163 was shown to be a significant negative prognosticator for patients with cancers of the breast, skin, head, and neck, among others [30,31,32,33]. Multiple manuscripts included in the current meta-analysis analyzed CD163 besides CD206, of which most manuscripts associated both markers with a negative prognosis [15,34,35,36]. Interestingly, two of the included manuscripts associated an increased CD206-expression with a significantly positive impact on the patients’ prognosis [17,18]. This ambiguity emphasizes the potential importance of additional unidentified prognosticators interacting with the M2-macrophages. Identifying the ‘ideal’ marker, or rather a combination of markers, for protumorigenic macrophages, might therefore be of interest to further characterize the prognostic impact of TAMs.

As stated above, TAMs cannot easily be differentiated in the protumorigenic M2 and antitumorigenic M1 but are rather part of a spectrum characterized by the presence of the overarching macrophage-specific CD68. While CD68 cannot be used as an independent prognosticator, the relative presence of the pro- or antitumorigenic markers, over the total amount of TAMs has shown to be a more reliable prognosticator than the sole marker itself [37,38]. In the manuscripts included in this review, multiple authors found a (more) significant result when a ratio of CD206^+^-cells over the total amount of CD68^+^ TAMs was analyzed, over CD206 alone [26,39,40]. Moreover, multiple authors stated an inverse correlation between the presence of CD206 and the M1-marker CD86, where CD206^high^ and CD86^low^-expression were associated with a significantly worse survival as compared to that of CD206^low^CD86^high^ [41,42,43,44]. It can therefore be hypothesized that is not the total amount of TAMs or M2-macrophages that have an impact on the survival, but rather the ratio of anti- (M1) and protumorigenic (M2) macrophages over the total amount of macrophages that could be independent prognosticators for the oncological patient’s survival [45,46,47]. The ratio of M1/M2 TAMs over the total amount of CD68+ macrophages could hence differentiate between a good (M1^high^ M2^low^) and poor (M1^low^ M2^high^) prognosis.

TAMs are only a small part of a more complex immune response. The interaction of TAMs and tumor infiltrating lymphocytes (TILs) results in a tumor-specific heterogeneous immune fingerprint, a process which might impact the therapeutic approach [48,49]. This hypothesis was confirmed by multiple reports stating that CD206-expression was correlated with the possible impact on different non-surgical therapeutic approaches, including chemotherapy and immunotherapy [50,51,52,53]. Consequentially, the presence of CD206-expressing macrophages in the tumor microenvironment could be used as a possible tool to predict tumor response to different therapies [54]. However, as different therapeutic approaches target different steps in the oncological process, the interaction between CD206 and the different approaches should individually be analyzed.

Although the current systematic review and meta-analysis provides additional information on the value of tumor-associated macrophages in solid cancers, we do acknowledge some limitations. Because we included the data of patients diagnosed with all types of solid tumors, independent of therapy, stage or histology, we collected a variable amount of definitions, outcomes, and methodologies, resulting in an important heterogeneity. Despite this heterogeneity, we demonstrate an important impact of CD206-expression on the OS in several solid tumor types. A different limitation is the fact that most of the studies described in this review used data of patients undergoing primary surgical resection of the tumor. While this includes an important cohort, a large cohort of patients undergoing nonsurgical therapy such as radiotherapy and/or systemic therapy was missed. As a change of CD206-expression is also expected in these patients, future research is needed to further explore the impact of different therapeutic approaches on the expression of TAMs.

## 5. Conclusions

In the current systematic review and meta-analysis, we demonstrated that the increased presence of CD206-expressing macrophages has a significant impact on the oncological outcome in patients with multiple types of solid cancers. These results strengthen the importance of characterizing the immune cells in the tumor microenvironment to predict and tackle the oncological patient’s prognosis. While additional research is needed, CD206 could be used to alter the therapeutic strategy towards a more patient-tailored approach. 

## Appendix A

**Table A1:** PubMed search string.

1	“mannose receptor”[tiab] OR CD206[tiab] OR “Cluster of Differentiation 206”[tiab] OR MRC1[tiab]
	3.378
2	MMR[tiab]
	7.766
3	Measles[tiab] OR mumps[tiab] OR Rubella[tiab] OR “major molecular response”[tiab] OR “mismatch repair”[tiab] OR “mismatched repair”[tiab] OR “maternal mortality rate”[tiab] OR “maternal mortality ratio”[tiab] OR “Mass miniature radiography”[tiab] OR “major molecular response”[tiab] OR “major molecular responses”[tiab] OR “major molecular remission”[tiab] OR “mediastinal mass ratio”[tiab] OR “midline malignant reticulosis”[tiab] OR “mass miniature radiophotography”[tiab]
	48.557
4	Prognosis[Mesh] OR Prognos *[tiab] OR Survival[mesh] OR surviv *[tiab] OR “Survival Analysis”[Mesh] OR hazard[tiab] OR “disease-free”[tiab] OR “disease free”[tiab] OR progressionfree[tiab] OR “progression free”[tiab] OR Kaplan-meier[tiab] OR “Kaplan meier”[tiab] OR predict *[tiab] OR “Outcome Assessment (Health Care)”[Mesh] OR outcome[tiab] OR efficacy[tiab] OR effective *[tiab]OR “Recurrence”[Mesh] OR recur *[tiab] OR relaps *[tiab] OR recrudesce *[tiab] OR (late[tiab] AND (effect[tiab] OR effects[tiab] OR complication *[tiab] OR onset[tiab])) OR sequela *[tiab] OR “long term”[tiab] OR longterm[tiab] OR following[tiab] OR “follow up”[tiab] OR followup[tiab] OR surviv *[tiab] OR “Survivors”[Mesh] OR mortality[tiab] OR “Longitudinal Studies”[Mesh] OR Time factors[MeSH] OR “treatment outcome”[tiab] OR Complications[tiab] OR Risk factors[MeSH]
	9.622.063
5	Neoplasms[Mesh] OR tumor *[tiab] OR cancer *[tiab] OR neoplas *[tiab] OR malignanc *[tiab] OR melanoma *[tiab] OR *sarcoma[tiab] OR *carcinoma[tiab] OR *glioma[tiab]
	4.164.738
6	(#1 OR (#2 NOT #3)) AND #4 AND #5
	579

*: statistical significant result.

**Table A2:** Embase search string.

1	‘mannose receptor’:ti,ab,kw OR CD206:ti,ab,kw OR ‘Cluster of Differentiation 206′:ti,ab,kw OR MRC1:ti,ab,kw
	5.832
2	MMR:ti,ab,kw
	13.557
3	Measles:ti,ab,kw OR mumps:ti,ab,kw OR Rubella:ti,ab,kw OR ‘major molecular response’:ti,ab,kw OR ‘mismatch repair’:ti,ab,kw OR “mismatched repair”:ti,ab,kw OR “maternal mortality rate”:ti,ab,kw OR “maternal mortality ratio”:ti,ab,kw OR “Mass miniature radiography”:ti,ab,kw OR “Mass miniature radiography”:ti,ab,kw OR “major molecular response”:ti,ab,kw OR “major molecular responses”:ti,ab,kw OR “major molecular remission”:ti,ab,kw OR “mediastinal mass ratio”:ti,ab,kw OR “midline malignant reticulosis”:ti,ab,kw OR “mass miniature radiophotography”:ti,ab,kw
	59.219
4	Prognosis/exp OR Prognos *:ti,ab,kw OR Survival/exp OR surviv *:ti,ab,kw OR ‘Survival Analysis’/exp OR hazard:ti,ab,kw OR ‘disease-free’:ti,ab,kw OR ‘disease free’:ti,ab,kw OR progressionfree:ti,ab,kw OR ‘progression free’:ti,ab,kw OR Kaplan-meier:ti,ab,kw OR ‘Kaplan meier’:ti,ab,kw OR predict *:ti,ab,kw OR ‘Outcome Assessment (Health Care)’/exp OR outcome:ti,ab,kw OR efficacy:ti,ab,kw OR effective *:ti,ab,kw OR ‘Recurrence’/exp OR recur *:ti,ab,kw OR relaps *:ti,ab,kw OR recrudesce *:ti,ab,kw OR (late:ti,ab,kw AND (effect:ti,ab,kw OR effects:ti,ab,kw OR complication *:ti,ab,kw OR onset:ti,ab,kw)) OR sequela *:ti,ab,kw OR ‘long term’:ti,ab,kw OR longterm:ti,ab,kw OR following:ti,ab,kw OR ‘follow up’:ti,ab,kw OR followup:ti,ab,kw OR surviv *:ti,ab,kw OR ‘Survivors’/exp OR mortality:ti,ab,kw OR ‘Longitudinal Studies’/exp OR ‘Time factors’/exp OR ‘treatment outcome’:ti,ab,kw OR Complications:ti,ab,kw OR ‘Risk factors’/exp
	11.822.881
5	Neoplasms/exp OR tumor *:ti,ab,kw OR cancer *:ti,ab,kw OR neoplas *:ti,ab,kw OR malignanc *:ti,ab,kw OR melanoma *:ti,ab,kw OR sarcoma:ti,ab,kw OR carcinoma:ti,ab,kw OR glioma:ti,ab,kw
	5.644.376
6	(#1 OR (#2 NOT #3)) AND #4 AND #5
	1.819

*: statistical significant result.

**Table A3:** Web of Science search string.

1	TS = “mannose receptor” OR TS = “CD206” OR TS = “Cluster of Differentiation 206” OR TS = “MRC1”
	4.762
2	TS = “MMR”
	9.274
3	TS = ”Measles” OR TS = “mumps” OR TS = “Rubella” OR TS = “major molecular response” OR TS = “mismatch repair” OR TS = “maternal mortality rate” OR TS = “maternal mortality ratio” OR TS = “Mass miniature radiography” OR TS = “Mass miniature radiography” OR TS = “Mass miniature radiography” OR TS = “major molecular response” OR TS = “major molecular responses” OR TS = “major molecular remission” OR TS = “mediastinal mass ratio” OR TS = “midline malignant reticulosis” OR TS = “mass miniature radiophotography”
	50.964
4	TS = “Prognosis” OR TS = “Prognos *” OR TS = “Survival” OR TS = “surviv *” OR TS = “Survival Analysis” OR TS = “hazard” OR TS = “disease-free” OR TS = “disease free” OR TS = “progressionfree” OR TS = “progression free” OR TS = “Kaplan-meier” OR TS = “Kaplan meier” OR TS = “predict *” OR TS = “Outcome Assessment (Health Care)” OR TS = “outcome” OR TS = “efficacy” OR TS = “effective*” OR TS = “Recurrence” OR TS = “recur *” OR TS = “relaps *” OR TS = “recrudesce *” OR TS = “(late:ti,ab,kw AND (effect” OR TS = “effects” OR TS = “complication *” OR TS = “onset:ti,ab,kw)) OR sequela *” OR TS = “long term” OR TS = “longterm” OR TS = “following” OR TS = “follow up” OR TS = “followup” OR TS = “surviv *” OR TS = “Survivors” OR TS = “mortality” OR TS = “Longitudinal Studies” OR TS = “Time factors” OR TS = “treatment outcome” OR TS = “Complications” OR TS = “Risk factors”
	16.167.705
5	TS = “Neoplasms” OR TS = “tumor *” OR TS = “cancer *” OR TS = “neoplas *” OR TS = “malignanc *” OR TS = “*melanoma *” OR TS = “* sarcoma” OR TS = “* carcinoma” OR TS = “glioma”
	3.836.044
6	(#1 OR (#2 NOT #3)) AND #4 AND #5
	772

*: statistical significant result.

**Table A4:** Characteristics of included studies.

Cancer Type	Manuscript	Country	Type	HR	HR	CI	# Patients	Analysis Technique	Antibody Clone	Method of Scoring
Breast Cancer										
1	Koru-Sengul et al. [55]	USA	Breast cancer	OS	1.44	0.99–2.09	145	IHC	NA	Qual.
				PFS	1.65	1.16–2.35				
Colo(rectal) cancer										
2	Feng et al. [52]	China	Colon cancer	OS ^†^	3.69	2.30–5.91	521	IHC	5C11	Quant.
				DFS ^†^	2.93	1.99–4.31				
3	Ding et al. [36]	China	Colorectal cancer	OS ^†^	71.24	1.38–3685.26	73	IHC	D-1	Quant.
4	Strasser et al. [56]	Austria	Colorectal cancer	OS (multi)	0.79	0.53–1.18	29	IHC	19.2	Quant.
				TTR	1.87	1.07–3.29				
Ovarian cancer										
5	Le Page et al. [40]	Canada	Epithelial ovarian cancer	/			180	IHC&IF	5C11	Quant.
6	Zhang et al. [57]	China	High grade serous ovarian cancer	OS ^†^	1.70	1.04–2.78	150	IHC	NA	Qual.
Esophageal cancer										
7	Yamamoto et al. [51]	Japan	Esophagal cancer	OS ^‡^	2.9	1.30–7.8	86	IHC	D-1	Quant.
				OS multi ^‡^	3.0	1.2–7.3				
Gastric cancer										
8	Liu et al. [34]	China	Gastric cancer	OS ^§^	2.27	1.38–3.72	120	IHC	polyclonal	Qual.
				OS (multi) ^§^	1.98	1.18–3.34				
				DFS ^§^	2.45	1.18–3.30				
				DFS (multi) ^§^	2.58	1.54–3.49				
9	Fu et al. [35]	China	Gastric cancer	OS ^†,§^	1.54	1.00–2.37	36	IHC	Abcam clone NA	Quant.
10	Zhang et al. [39]	China	Gastric cancer	OS	1.71	1.13–2.6	180	IHC	Abcam clone NA	Quant.
Glioma										
11	Ding et al. [43]	China	Glioma	OS ^†^	3.52	3.52–3.52	50	IHC	NA	Quant.
				PFS ^†^	10.47	10.47–10.47				
Hepatocellular carcinoma										
12	Dong et al. [42]	China	Hepatocellular Carcinoma	OS (multi)	1.58	1.05–2.39	253	IHC	Abcam clone NA	Quant.
				TTR (multi)	1.87	1.07–3.29				
13	Fan et al. [58]	China	Hepatocellular Carcinoma	OS	1.84	1.23–2.75	327	IHC	ab64693	Qual.
14	Ren et al. [59]	China	Hepatocellular Carcinoma	OS (multi)	1.79	1.12–2.88	268	IHC	ab117644	Quant.
				DFS (multi)	2.22	1.28–3.86				
15	Shu et al. [60]	China	Hepatocellular Carcinoma	OS	0.45	0.26–0.76	80	IHC	ab64693	Quant.
				OS (multi)	1.94	1.06–3.55				
16	Zhu et al. [61]	China	Hepatocellular Carcinoma	OS	2.37 ^¶^	1.06–5.31	90	IHC	Abcam clone NA	Qual.
Cholangiocarcinoma										
17	Sun et al. [41]	China	Intrahepatic cholangiocarcinoma	OS	1.55	1.16–2.07	322	IHC	Abcam clone NA	Quant.
				PFS	1.42	1.05–1.91				
Head and neck cancers										
18	Ooft et al. [14]	Netherlands	Nasopharyngeal carcinoma	/	NA	NA	91	IHC	1C9	Quant.
19	Haque et al. [62]	Japan	Oral squamous cell carcinoma	DFS	3.29 ^†^	1.1–14.1	44	IHC	5C11	Quant.
				PFS	3.28 ^†^	1.1–14.1				
20	Weber et al. [15]	Germany	Oral squamous cell carcinoma	/			34	IHC	5C11	Quant.
Pancreatic cancer										
21	Di Caro et al. [16]	Italy	PDAC	/			32	IHC	755339	Quant.
22	Hu et al. [26]	China	PDAC	OS	1.861	1.16–2.99	77	IHC	NA	Qual.
				OS (multi)	1.595	0.99–2.57				
23	Mahajan et al. [18]	Germany	PDAC	PFS	0.64	0.47–0.87	385	IHC	685645	Quant.
Penile cancers										
24	Chu et al. [17]	China	Penile cancer	DSS	0.349	0.19–0.63	178	IHC	Ab64693	Quant.
Prostate cancer										
25	Hu et al. [63]	China	Prostate Ca	OS	1.18	1.09–1.28	42	IF	ab64693	Quant.
				OS (multi)	0.84	0.68–1.04				
Renal cell carcinoma										
26	Xu et al. [44]	China	Renal cell carcinoma	DSS	1.949	1.11–3.42	185	IHC	Abcam clone NA	Quant.
Melanoma										
27	Enninga et al. [64]	USA	Stage IV Melanoma	OS ^†^	2.37	1.15–4.87	180	IHC	685645	Quant.

CI: confidence interval, the CI’s described here are those given by the authors, or calculated based on the author’s survival analysis rounded to two decimals. †: value was calculated based on the Kaplan Meier curve. ‡: Odds ratio was given and CD206+ and CD168+ were used to differentiate survival analysis; multi: multivariable analysis; OS: overall survival; PFS: progression-free survival; DFS: disease-free survival; DSS: disease-specific survival; IOD: integrated optical density sum; §: Only CD163+CD206+ data was given; ¶: Relative risk was given.
